# Relationship Between Knowledge, Attitudes, and Practices for the Consumption of *Spirulina*-Enriched Fruit and Vegetable Juices: Structural Equation Modelling and Consumers’ Preference Evaluation Approach

**DOI:** 10.3390/nu18081309

**Published:** 2026-04-21

**Authors:** Miona Belović, Lato Pezo, Goran Radivojević, Mirjana Penić, Jasmina Lazarević, Bojana Filipčev, Uroš Čakar, Jasmina Vitas, Biljana Cvetković

**Affiliations:** 1 Institute of Food Technology, University of Novi Sad, Bulevar cara Lazara 1, 21000 Novi Sad, Serbia; jasmina.lazarevic@fins.uns.ac.rs (J.L.); bojana.filipcev@fins.uns.ac.rs (B.F.); biljana.cvetkovic@fins.uns.ac.rs (B.C.); 2 Institute of General and Physical Chemistry, Studentski trg 12/V, 11000 Belgrade, Serbia; latopezo@yahoo.co.uk; 3 Faculty of Sciences, University of Novi Sad, Trg Dositeja Obradovića 3, 21000 Novi Sad, Serbia; goran.radivojevic@dgt.uns.ac.rs (G.R.); mirjana.penic@dgt.uns.ac.rs (M.P.); 4 Department of Bromatology, Faculty of Pharmacy, University of Belgrade, Vojvode Stepe 450, 11221 Belgrade, Serbia; uros.cakar@pharmacy.bg.ac.rs; 5 Faculty of Technology, University of Novi Sad, Bulevar cara Lazara 1, 21000 Novi Sad, Serbia; vitasj@uns.ac.rs

**Keywords:** *Spirulina*, enriched juice, survey, behavioural intention, structural equation modelling, consumer preferences

## Abstract

**Background/Objectives:** The presented study aimed to understand the relationship between knowledge, attitudes, and practices, as well as consumers’ preferences for the consumption of *Spirulina*-enriched fruit and vegetable juices. **Methods:** A survey about the consumers’ attitudes towards consumption of algae in general and especially *Spirulina* was conducted to better understand the target groups and marketing strategies for this novel non-alcoholic beverage product. Knowledge–Attitude–Practice (KAP) model in combination with structural equation modelling (SEM) was applied to test the hypothesised relationships between the variables. Additionally, consumers’ preference test was done using a seven-point hedonic scale and ranking of the six juice samples: plain sour cherry juice (SC1), sour cherry juice with 0.8% (SC2) and 1.6% (SC3) of blue *Spirulina* powder; plain tomato juice (T1), tomato juice with 0.8% (T2) and 1.6% (T3) of blue *Spirulina* powder. **Results:** The SEM results showed that there is a limited direct impact of knowledge on social motivation, while personal behaviour strongly predicts social motivation. Namely, perceived nutritional value and health benefits were shown to be the main factors for consumers’ willingness to drink *Spirulina*-enriched juice. **Conclusions:** The result of the consumer preference evaluation exposed that the juices containing sour cherry and *Spirulina* achieved better sensory acceptance and ranking than those containing tomato, pointing out the importance of the product matrix for achieving consumer acceptance.

## 1. Introduction

The food system transition implies increasing plant-based and alternative proteins, driven by environmental and social concerns [1]. In the recent years, algae have attracted increasing attention as functional food ingredients due to their high nutritional value, sustainability, and potential health benefits. Among them, *Spirulina* (*Arthrospira platensis*) has been widely recognized as a “superfood” because of its high protein content (50–60% of dry weight), essential amino acids (prominently γ-linolenic acid), vitamins (especially B_12_, which is deficient in plant-based diets), minerals (mainly iron), and bioactive compounds [2,3,4]. It is important to note that the bioavailability of vitamin B_12_ for humans depends on the cultivation conditions. Common *Spirulina* supplements typically contain the inactive form, known as pseudovitamin B_12_ (cobamide) [5]. The incorporation of *Spirulina* into the human diet has been linked to positive health outcomes, such as the prevention of malnutrition, antioxidant activity, and immune support [6,7]. *Spirulina*, as a climate-resilient food source, can help address food security challenges caused by climate change due to its low resource requirements and rapid growth [8].

Despite these promising properties, consumer acceptance of algae-based products remains a critical factor for their successful integration into everyday nutrition. Perceptions of taste, appearance, price, and familiarity with algae are known to strongly influence consumer attitudes and purchasing decisions. Namely, food neophobia, defined as the tendency for consumers to avoid consumption of more “novel” foods was identified as a significant barrier to consumer acceptance of algae. Moreover, macroalgae, usually called seaweeds, have been shown to be more acceptable due to the familiarity of consumers with sushi [9]. Generally, familiarity with the consumed product largely determines the attitude of consumers. Namely, Grahl et al. [2] studied consumer’s willingness to try three innovative products: pasta filled with *Spirulina*, maki-sushi filled with *Spirulina*, and *Spirulina* jerky. As the most familiar product, pasta filled with *Spirulina* was the most preferred product. Similarly, meat substitute prepared from micro-algae was appealing mostly to consumers that are willing to try meat substitutes [10]. There is not much data on the flavour contribution of microalgae to plant-based food alternatives [3]. In general, freshwater algae, such as *Arthrospira platensis*, were evaluated as tastier than the marine algae due to the lack of “fishy” taste characteristic for marine species [11].

Generally, algae, including *Spirulina*, were used to enrich cereal, dairy and meat products, while their application to vegetable and fruit products remain limited on broccoli soup and green smoothie [12,13]. However, previous studies have shown that 85% of participants were open to consuming algae, especially in familiar products such as bread, pasta, biscuits and juices that reduce food neophobia [4,14]. However, in contrast to foods, beverages have a tendency to expose novel ingredients, which provides an appeal to neophilic consumers and a challenge to neophobic consumers [15]. Researchers are primarily interested in fruit juices for their health benefits, perceiving them as a simple and effective vehicle for nutrients [16]. Additionally, novel juice formulations that emphasize natural ingredients, health benefits, and balanced sensory profiles are well-received by consumers [17]. Following this information, the previous study performed by our research group dealt with the nutritional and sensory analyses of juices enriched with blue *Spirulina* powder [18]. The sour cherry and tomato juices have been selected after the preliminary sensory assessment by a trained panel for the addition of blue *Spirulina* powder. We hypothesized that, despite the promising results of sensory analysis performed by a trained panel, consumers’ knowledge, attitudes, and practices, as well as their preferences should be evaluated since products with microalgae have not yet penetrated the market [19].

Structural equation modelling (SEM) was applied to examine the interrelationships among knowledge, attitudes, and practices within the Knowledge–Attitude–Practice (KAP) framework [20]. The KAP model assumes that knowledge shapes attitudes, which in turn influence practices, while allowing for potential direct effects of knowledge on behaviour [21]. The KAP model is particularly useful for assessing novel *Spirulina* products because it links consumer awareness and understanding (knowledge) to perceptions (attitudes) and actual consumption behaviour (practice). This framework allows researchers to identify specific barriers to acceptance, such as unfamiliar taste or perceived health concerns, and to design interventions that can improve adoption of algae-based foods. SEM is particularly suitable for this purpose as it enables the simultaneous estimation of latent constructs and their structural relationships [22], providing a comprehensive assessment of both measurement quality and hypothesised causal pathways, related to *Spirulina* incorporation into fruit and vegetable juices. Moreover, SEM was used in the studies dealing with the intention of consumers in Ethiopia to consume *Spirulina*-fortified bread [23] and in the analysis of purchase intentions and willingness to pay for microalgae products [24].

In addition to the KAP framework, a consumer survey was conducted to assess general perceptions of algae and *Spirulina* acceptability, complemented by sensory evaluation outcomes. Penalty analysis summary statistics were applied to identify attributes driving consumer dissatisfaction [25], while mean liking scores after assessor centring were used to obtain a more reliable comparison of sensory preferences.

## 2. Materials and Methods

### 2.1. Preparation of Juices

The juices were prepared as described before in the paper by Cvetković et al. [18]. Namely, “Oblačinska” variety of sour cherry (*Prunus cerasus* L.), and tomato (*Solanum lycopersicum* L.) variety for processing “Šljivar” were purchased from a local market, and the juices were prepared using a Bosch MES 3500 culinary juicer, BSH Home Appliances Ltd (Wolverton, UK). The blue *Spirulina* powder was purchased from NatureHub (Belgrade, Serbia; https://www.naturehub.rs/, accessed on 27 November 2025). It primarily consisted of phycocyanin extracted from *Arthrospira platensis*, as written in the manufacturer’s declaration. Blue *Spirulina* powder was added to each juice at two levels: 2 g (0.8% *w*/*w*) and 4 g (1.6% *w*/*w*) per 250 mL serving (one glass of juice). Six samples were prepared in total: plain sour cherry juice (SC1), sour cherry juice with 0.8% (SC2) and 1.6% (SC3) of blue *Spirulina* powder; plain tomato juice (T1), tomato juice with 0.8% (T2) and 1.6% (T3) of blue *Spirulina* powder. After adding the powder, the juice samples were homogenized using an Ultra-Turrax T25 homogenizer (IKA, Staufen, Germany), operating at 10,000–13,000 rpm for 1–2 min to ensure complete dispersion of the powder in the juice.

### 2.2. Consumers’ Survey on Algae and Spirulina Acceptability

A consumer survey was conducted to assess perceptions, attitudes, and potential acceptance of algae in human nutrition, with a particular focus on *Spirulina*. A total of 136 respondents participated in the study. The questionnaire consisted of demographic questions (gender, age, education, employment status, and income level) as well as items designed to capture awareness, knowledge, attitudes, and behavioural intentions regarding algae consumption (Supplementary File S1). To evaluate respondents’ opinions, a five-point Likert scale (1 = strongly disagree, 5 = strongly agree) was employed, allowing for nuanced measurement of agreement with statements relating to nutritional value, health benefits, sustainability, affordability, and social influence on algae consumption. This design enabled both descriptive and inferential statistical analysis of consumer perceptions, providing insights into potential drivers and barriers to incorporating algae-based products into everyday diets.

The data was recorded via Google Forms on the cloud, and was exported in the form of a Microsoft Excel document, using the CAWI methodology (Computer-assisted web interviewing). This method uses software to conduct surveys or interviews via web platforms, providing more efficient responses via computer, tablet or smartphone, as well as collecting and processing completed surveys based on CAWI methods. The required time to fill out the questionnaire was 5–10 min. The research was anonymous, and by filling out the questionnaire, respondents give their consent to participate. Respondents could withdraw from the research at any time, which will be noted to them before filling out the questionnaire. Model fit was evaluated using the Comparative Fit Index (CFI), Goodness-of-Fit Index (GFI), Normed Fit Index (NFI), and the Root Mean Square Error of Approximation (RMSEA), with missing data handled by list-wise deletion.

### 2.3. Sensory Evaluation Design

Sensory evaluation was conducted using a consumer-based approach to assess the acceptability of six product samples, coded as SC1–SC3 and T1–T3. 110 willing volunteers (ages 18–56; 63 females and 47 males) were chosen based on their willingness and lack of aversion for the substances used to prepare the samples. The survey (*n* = 136) and sensory evaluation (*n* = 110) were conducted on two independent and non-overlapping consumer samples. Each respondent verbally consented to take part in the assessment and stated that they had no known food allergies, specifically to the juices used (sour cherry and tomato) and to *Spirulina*, in addition to general food safety considerations. They were asked to rate their preference of six juice samples in terms of colour, odour, texture, and taste, as well as to assess their overall acceptability using a seven-point hedonic scale (from 1 = extremely dislike to 7 = extremely like). A total of 110 valid observations were collected for each sensory attribute, with no missing data, ensuring completeness and robustness of the dataset. Additionally, the ranking score across all samples was summed up to provide an overall indication of consumer preference distribution [26]. The juice samples were arranged in a random order after being coded with random three-digit numbers. Tests were conducted in a controlled sensory laboratory setting [27].

### 2.4. Penalty Analysis

A just-about-right (JAR) scale-based penalty analysis was applied to evaluate the impact of deviations in sensory attributes on overall hedonic perception of the product. Respondents evaluated colour, odour, texture, taste, and overall acceptability using a structured JAR scale consisting of three categories: “too little,” “just-about-right (JAR),” and “too much.” Hedonic ratings were collected in parallel using a 7-point hedonic scale.

Penalty analysis was performed by comparing mean hedonic scores across JAR deviation categories. The mean drop was calculated as the difference between the mean hedonic score of the JAR group and the respective “too little” or “too much” group. In addition, standardized differences and associated *p*-values were computed to assess the statistical significance of penalties associated with deviations from the optimal (JAR) level. This approach allowed identification of potential sensory drivers of liking and quantification of the effect of suboptimal attribute intensity on consumer acceptability.

### 2.5. Statistical Analysis

Descriptive statistics (means, standard deviations, and rank distributions) were used to summarize sensory data and assess variability in consumer responses. Comparisons between product groups (SC vs. T) were based on differences in mean hedonic scores and response dispersion across attributes. All analyses were conducted on complete datasets to avoid bias due to missing values.

Questionnaire data were analysed in R software (version 4.4.3) using a structural equation modelling (SEM) framework to evaluate knowledge, attitudes, and practices (KAP). SEM, as an extension of multiple regression, was applied to simultaneously assess measurement models and structural relationships among latent constructs defined by questionnaire sections [28], with individual items serving as observed indicators.

Penalty analysis was applied to identify sensory attributes that negatively influenced overall acceptability [29]. This approach allowed the quantification of “penalties” associated with attributes receiving lower hedonic ratings, particularly when deviations from optimal perception resulted in reduced overall liking. The analysis focused on detecting attributes with substantial negative impact on consumer acceptance and on comparing the magnitude of these penalties between SC and T product series.

## 3. Results

### 3.1. Survey Analysis

Inspection of the Likert-scale questionnaire items (Q1–Q22) (Table 1) confirmed complete data integrity [30]. No missing values were detected for any item, as all variables exhibited zero missing observations. In addition, examination of the observed response ranges demonstrated full compliance with the intended five-point Likert scale, with all items spanning the complete range from 1 to 5.

These results indicate correct data coding, absence of non-numeric or out-of-scale values, and full utilization of the response scale by participants. Consequently, the dataset is suitable for subsequent statistical analyses, including correlation analysis and structural equation modelling, without the need for imputation or data transformation [31].

### 3.2. Descriptive Statistics of the Survey Data

The socio-demographic structure of the sample (*n* = 136) indicates a predominance of female respondents, who accounted for 71.32% of the participants, while males represented 28.68%. This gender imbalance suggests that the findings may be more reflective of female consumer attitudes and preferences. The age distribution of respondents was relatively broad, ranging from 19 to 72 years, indicating a heterogeneous sample. However, a higher concentration of participants was observed in the young adult and middle-aged groups, particularly between 20 and 50 years of age. The cumulative distribution shows that approximately 52% of respondents were aged 40 years or younger, while about 76% were below 50 years, suggesting a slight predominance of a younger and economically active population.

In terms of educational level, the sample was relatively well-educated. The majority of respondents (58.82%) held a university degree, followed by those with secondary education (32.35%), while a smaller proportion had completed higher vocational education (8.09%). Only one respondent (0.74%) reported primary education. This indicates that the sample is skewed toward individuals with higher educational attainment, which may influence knowledge and attitudes toward alternative food products.

The study employed a convenience sampling approach based on voluntary participation, which is frequently applied in exploratory consumer studies. As a consequence, the sample exhibited a higher proportion of female respondents and participants with university-level education. Such sample characteristics are typical for non-probability sampling designs and should be considered when interpreting the generalizability of the results.

Regarding employment status, most respondents were employed (69.85%), followed by students (15.44%) and entrepreneurs (8.09%). Retirees (4.41%) and unemployed individuals (2.21%) were less represented. This distribution suggests that the sample is largely composed of economically active individuals, which may be relevant when interpreting purchasing behaviour and willingness to adopt new products.

With respect to income, respondents were relatively evenly distributed across categories: 34.56% reported above-average income, 32.35% average income, and 30.15% below-average income, while 2.94% of responses were missing. This relatively balanced distribution enhances the representativeness of the sample in terms of economic diversity.

Descriptive statistics (Table 2) for the Likert-scale items (Q1–Q22; *n* = 136) indicate moderate to high central tendency across most variables, with mean values ranging from 2.30 (Q5) to 4.07 (Q3) and median scores predominantly at 3 or 4. Items Q3, Q2, Q1, Q14, Q18, and Q21 exhibited the highest mean ratings (≥3.66), reflecting generally positive evaluations perceptions of *Spirulina*, regarding its nutritional value, health benefits, and potential to reduce disease risk, whereas Q5 (mean = 2.30) and Q17 (mean = 2.33) showed comparatively lower agreement. The low mean score for Q5 indicates that participants generally lack knowledge on how to prepare *Spirulina* for consumption. This supports the notion that practical knowledge is a key barrier to acceptance, as consumers may be aware of *Spirulina*’s benefits but feel uncertain about how to use it in foods, highlighting the need for clear preparation guidelines in product development.

Standard deviations ranged from 1.17 (Q3) to 1.69 (Q6), indicating moderate response dispersion typical of five-point Likert data. All items demonstrated non-zero variance (range: 1.36–2.86), confirming sufficient variability and ruling out floor or ceiling effects that could compromise subsequent multivariate analyses. The highest variance was observed for Q6 (2.86), suggesting greater heterogeneity in responses for this item.

Distributional shape measures revealed mild to moderate skewness across items (skewness: −1.03 to 0.60), with negatively skewed distributions predominating, indicating a tendency toward higher ratings. Kurtosis values were generally negative (–1.63 to 0.08), suggesting flatter-than-normal distributions consistent with ordinal, bounded response scales.

The descriptive statistics confirm adequate central tendency, dispersion, and variability across all questionnaire items, supporting their suitability for correlation analysis and structural equation modelling without the need for item exclusion or transformation [32,33]. Overall, respondents demonstrated a relatively high level of awareness regarding the nutritional and health benefits of algae, including *Spirulina*, as reflected by higher mean scores for items related to perceived nutritional value and health relevance. Similar results were obtained in survey in Spanish population, where microalgae were considered nutritious and healthy [14]. In Italian population, young, physically active, well-educated men, interested in healthy eating and open to trying new foods represent the target population for *Spirulina*-enriched foods [19].

### 3.3. Normality Assumptions

Univariate normality of the Likert-scale items (Q1–Q22) was assessed using the Kolmogorov–Smirnov test applied to standardized scores. For all variables, the test yielded statistically significant results, with *p*-values well below the *p* < 0.05 threshold (range: 1.09 × 10^−11^ to 2.80 × 10^−3^), leading to rejection of the null hypothesis of normality for every item.

The extremely small *p*-values observed for most variables (often <10^−6^) indicate pronounced departures from Gaussian distributions rather than marginal deviations. These findings are consistent with the ordinal, bounded nature of five-point Likert-scale data and with the skewness and kurtosis patterns observed in the descriptive statistics.

Normality assessment using the Shapiro–Wilk and Anderson–Darling tests [34] indicated significant departures from normality for all 22 variables (Q1–Q22), with *p*-values < 0.05 in both tests and frequently *p* < 10^−8^, leading to rejection of the normality assumption across all attributes. Given the ordinal and bounded nature of the data, non-parametric statistical methods were therefore applied.

Levene’s test showed that variance homogeneity [35] was largely satisfied across assessor blocks (A1–A5), with non-significant results (*p* > 0.05) for the majority of attributes. Only a few isolated assessor–attribute combinations exhibited heteroscedasticity, namely A4–Q2 (*p* = 0.011), A4–Q22 (*p* = 0.040), and A5–Q10 (*p* = 0.009).

Group comparisons using the Mann–Whitney U test [36] revealed no statistically significant differences between the two independent groups for any attribute (all *p* > 0.05), with Q2 approaching but not reaching significance (*p* = 0.069), indicating overall group comparability.

The Kruskal–Wallis test across multiple groups [37] showed predominantly non-significant effects (*p* > 0.05), with only three significant assessor–attribute cases: A3–Q8 (*p* = 0.033), A4–Q3 (*p* = 0.002), and A4–Q17 (*p* = 0.040). Several additional variables exhibited borderline *p*-values near 0.05, but these effects were isolated and not systematic.

These results indicate minimal and attribute-specific group effects, supporting data aggregation for subsequent analyses while acknowledging limited assessor-dependent variability.

### 3.4. Multivariate Normality

Multivariate normality and the presence of potential multivariate outliers were assessed using Mahalanobis distance [38] based on the covariance structure of the Likert-scale items (Q1–Q22). Using a conservative χ^2^ cutoff corresponding to *p* < 0.001 with 22 degrees of freedom, two observations exceeded the critical value and were identified as potential multivariate outliers.

The very small number of detected outliers (2 out of 136 observations; 1.47%) indicates that extreme multivariate response patterns were rare and not pervasive in the dataset. Given the ordinal nature of the data and the subsequent use of non-parametric and robust analytical methods, these observations were not considered to represent systematic data anomalies.

For SEM analyses, four key assumptions were assessed: multivariate normality [39], multicollinearity [22], sample size adequacy [40], and positive definiteness [41].

### 3.5. Multicollinearity Diagnostics

Multicollinearity among the Likert-scale items (Q1–Q22) was evaluated using variance inflation factors (VIF) and tolerance values [28]. VIF values ranged from 1.60 (Q17) to 13.82 (Q12). While the majority of items exhibited acceptable levels of collinearity (VIF < 5), the elevated VIF observed for Q12 indicates a high degree of multicollinearity, suggesting substantial overlap with other health-related variables. This may reflect redundancy among conceptually similar items and should be considered when interpreting the factor structure and parameter estimates. Such elevated collinearity is not uncommon in latent variable models where indicators are designed to capture closely related aspects of the same underlying construct. Tolerance values correspondingly ranged from 0.07 (Q12) to 0.63 (Q17), with very low tolerance observed for Q12, Q20, and Q15, suggesting potential multicollinearity concerns for these specific items. These findings indicate that the dataset is largely suitable for multivariate analyses, such as structural equation modelling, though attention should be paid to highly collinear items during model specification or latent construct estimation.

### 3.6. Correlation

**Figure 1:**
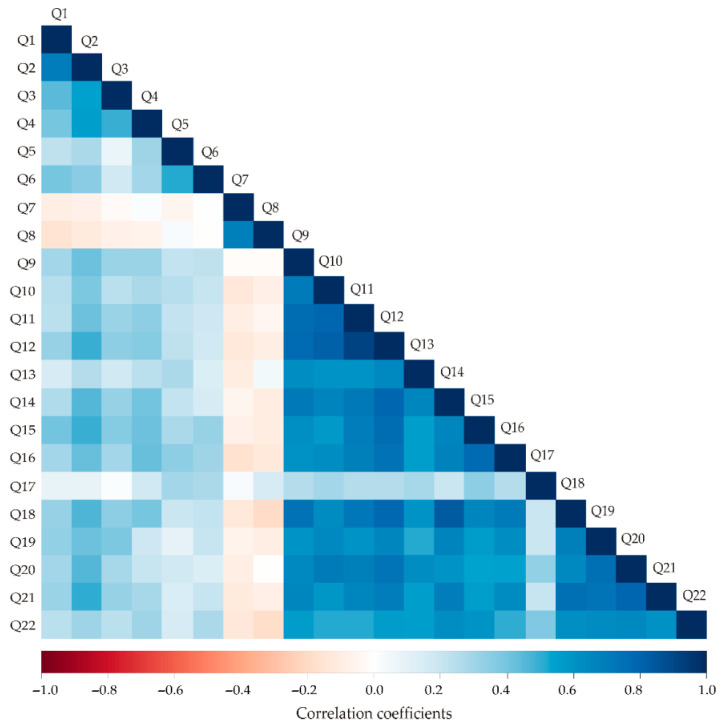
Colour correlation plot for Q1–Q22 variables.

To further explore the relationships between consumers’ knowledge, attitudes, intentions, and self-reported behaviour related to algae and *Spirulina* consumption, correlation analysis was performed. The Spearman correlation analysis (Figure 1) reveals numerous statistically significant associations among the variables (Q1–Q22) based on a sample size of 136.

Most correlations are positive and moderate to strong, with several exceeding 0.7, indicating high monotonic relationships. For instance, Q1 is strongly correlated with Q2 (r = 0.699, *p* < 0.001), moderately with Q3 (r = 0.440, *p* < 0.001) and Q4 (r = 0.397, *p* < 0.001), and weakly with Q5 (r = 0.221, *p* = 0.010) and Q10 (r = 0.237, *p* = 0.006). Q2 shows strong correlations with Q3 (r = 0.550, *p* < 0.001), Q4 (r = 0.556, *p* < 0.001), and moderate correlations with Q6 (r = 0.279, *p* = 0.001), Q8 (r = 0.357, *p* < 0.001), and Q10–Q12 (r = 0.383–0.491, *p* < 0.001). High correlations appear among several variables in the Q9–Q15 range, for example, Q9 correlates very strongly with Q10 (r = 0.795, *p* < 0.001), Q11 (r = 0.913, *p* < 0.001), and Q12 (r = 0.644, *p* < 0.001), indicating these may represent closely related constructs. Other notable correlations include Q14 with Q18 (r = 0.840, *p* < 0.001) and Q18 with Q19 (r = 0.755, *p* < 0.001), suggesting strong associations in this cluster. Some weaker but still significant relationships were observed, such as Q6 with Q5 (r = 0.223, *p* = 0.009) and Q17 with Q16 (r = 0.258, *p* = 0.002), highlighting minor associations.

The results indicate that many of the questionnaire items are interrelated, with several strong positive correlations exceeding 0.7, several moderate correlations between 0.3 and 0.6, and some weaker but significant associations below 0.3, suggesting both redundancy among some items and meaningful variability across others. These patterns could guide factor analysis or dimensionality reduction to identify underlying constructs in the dataset.

### 3.7. Kaiser-Meyer-Olkin and Bartlett’s Test of Sphericity

Sampling adequacy and factorability of the questionnaire items (Q1–Q22) were assessed using the Kaiser–Meyer–Olkin (KMO) measure and Bartlett’s test of sphericity [32] based on the Spearman correlation matrix. The overall KMO value was 0.89, indicating excellent sampling adequacy for factor analysis. Individual Measures of Sampling Adequacy (MSA) ranged from 0.48 (Q7) to 0.97 (Q9), with most items above 0.75, supporting their suitability for multivariate analysis. Bartlett’s test of sphericity was highly significant (χ^2^ = 2349.67, *df* = 231, *p* < 0.001), confirming that the correlation matrix was not an identity matrix and that the variables were sufficiently interrelated for factor extraction. These results indicate that the dataset is suitable for exploratory and confirmatory factor analyses, including structural equation modelling, as all SEM assumptions were satisfied [33].

### 3.8. Exploratory Factor Analysis

Multiple moderate to strong correlations between subsets of items indicate that respondents’ responses are structured around shared underlying dimensions rather than being independent. Thus, by classifying related questionnaire items into interpretable factors, exploratory factor analysis was used to find latent constructs and simplify the data. Exploratory factor analysis (EFA) with oblimin rotation and maximum likelihood extraction [42] was conducted on the 22 Likert-scale items. The EFA was chosen because the survey was designed to explore underlying dimensions of knowledge, attitudes, and practices toward *Spirulina*, rather than to test a pre-specified factor structure. This approach allows for the identification of latent constructs in a data-driven manner, which is appropriate for a novel product with limited prior research. Three factors were retained based on theoretical considerations and factor interpretability. Factor loadings ≥ 0.40 are shown in Table 3.

Factor 1 (ML1) accounted for 20.9% of the total variance and included items primarily related to personal intentions and behaviours regarding *Spirulina* consumption (Q9, Q10, Q11, Q12, Q13, Q14, Q15, Q16, Q18). Factor 2 (ML3) explained 13.5% of variance and comprised items reflecting social motivation and external influences on consumption (Q19, Q20, Q21, Q22). Factor 3 (ML2) captured 11.5% of variance and included knowledge-related items (Q1, Q2, Q3, Q4, Q6, Q9), indicating understanding of algae and their nutritional properties. Factor 1 (ML1) measures an individual’s personal intention and motivational attitude toward *Spirulina*, integrating both health beliefs and behavioural drivers. Factor 2 (ML3) corresponds to health concerns, which reflect awareness of algae in nutrition, health benefits, and prior consumption experience. Factor 3 (ML2) represents external and hedonic motivations for *Spirulina* consumption. It captures the influence of price, social approval, perceived health benefits, and sensory/novelty aspects on consumption in food service settings.

The cumulative variance explained by the three factors was 45.9%, suggesting that these dimensions capture a substantial proportion of variability in participants’ responses. Factor loadings were generally strong, with the highest loadings observed for Q11 (0.987) and Q12 (0.967), indicating robust contributions of these items to Factor 1.

The EFA results suggest a three-dimensional structure of the questionnaire, encompassing knowledge, personal intention/behaviour, and social motivation regarding *Spirulina* consumption, providing a meaningful framework for subsequent confirmatory analyses.

### 3.9. Reliability of Constructs

Reliability analysis [28] confirmed good to excellent internal consistency for all subscales, with Cronbach’s alpha values of 0.79 for Factor 1, 0.95 for Factor 2, and 0.92 for Factor 3, supporting the coherence of each factor. These results indicate that the questionnaire items form a meaningful, internally consistent three-dimensional structure suitable for subsequent confirmatory analyses and structural equation modelling. The reliability results indicate that all three factors derived from the EFA are generally consistent internally. Group 3 is highly reliable, Group 1 is acceptable with potential improvement by removing Q5, and Group 2 is moderately reliable but could benefit from the removal of Q7 or Q8 to strengthen consistency. These findings support the structural validity of the factors while highlighting specific items that may be less coherent with their respective scales.

### 3.10. Convergent Validity

Convergent validity [43] was assessed using Composite Reliability (CR) and Average Variance Extracted (AVE) for all latent constructs [32]. The CR values exceeded the commonly recommended threshold of 0.7 for all factors, indicating satisfactory internal consistency (F1: CR = 0.815; F2: CR = 0.953; F3: CR = 0.926). The AVE values, reflecting the proportion of variance captured by each construct relative to measurement error, were above 0.5 for F2 (AVE = 0.718) and F3 (AVE = 0.715), indicating adequate convergent validity, while F1 showed a slightly lower AVE (0.484), suggesting that although reliability is acceptable, the variance captured by the indicators for F1 is marginally below the preferred threshold. The results support acceptable convergent validity for the measurement model.

### 3.11. Discriminant Validity

Discriminant validity was evaluated using the Fornell–Larcker criterion [44], in which the square of latent variable correlations is compared with the Average Variance Extracted (AVE) of each construct. The squared correlations [28] between constructs were 0.321 for F1–F2, 0.358 for F1–F3, and 0.803 for F2–F3. Comparing these values with the AVE estimates (F1 = 0.484, F2 = 0.718, F3 = 0.715), all squared correlations were lower than the AVE of the respective constructs, indicating that each construct shares more variance with its own indicators than with other constructs. This confirms adequate discriminant validity of the measurement model [45], although the relatively high correlation between F2 and F3 (0.803) suggests some conceptual overlap between these factors.

### 3.12. SEM Analysis

**Figure 2:**
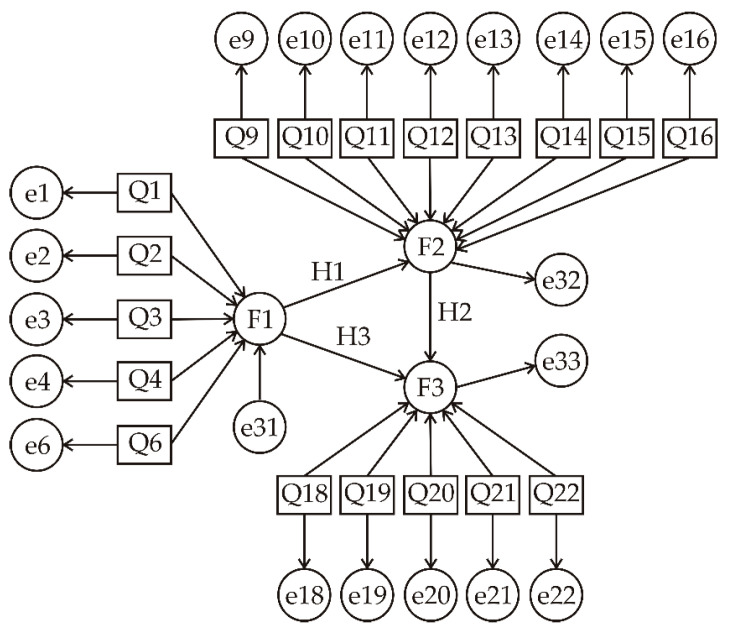
SEM analysis.

Utilizing the factor structure derived from exploratory factor analysis, structural equation modelling (SEM) was employed to investigate the correlations among consumers’ knowledge of algae and *Spirulina*, their intention to consume these products, and their self-reported consumption behaviour. A structural equation model (SEM) was specified to evaluate the relationships among the three latent factors identified by exploratory factor analysis: knowledge (F1), personal intention/behaviour (F2), and social motivation/influence (F3) (Figure 2). Based on the proposed conceptual framework and the SEM approach, three hypotheses were formulated at the latent construct level: knowledge (F1) has a positive effect on personal intention/behaviour (F2) (H1), while F2 positively influences social motivation/influence (F3) (H2). In addition, a direct positive effect of knowledge (F1) on social motivation/influence (F3) is assumed (H3), reflecting both indirect and direct pathways through which knowledge may shape behavioural and socially driven responses toward algae consumption. The measurement model included F1 measured by Q1, Q2, Q3, Q4, and Q6; F2 by Q9–Q16 (excluding Q7 and Q8); and F3 by Q18–Q22 (excluding Q17). The structural model specified that F2 is predicted by F1, and F3 is predicted by both F1 and F2.

The model was estimated using robust maximum likelihood (MLR) [46] with full information maximum likelihood handling of missing data. The model converged normally after 37 iterations. The chi-square test indicated a significant discrepancy between the model and the observed data (scaled χ^2^ = 335.64, *df* = 132, *p* < 0.001, scaled Yuan-Bentler correction) [47], which is common in models with moderate sample sizes and non-normal data.

The measurement model showed strong factor loadings for all observed indicators, with standardized loadings ranging from 0.421 (Q6) to 0.969 (Q12), indicating that the questionnaire items reliably represent their respective latent factors. Reliability was further supported by the proportion of explained variance (R^2^), with individual items ranging from 0.177 to 0.938 and the latent constructs showing R^2^ = 0.321 for F2 and R^2^ = 0.814 for F3.

The structural paths among the latent constructs indicate that knowledge (F1) significantly predicts personal intention/behaviour (F2), with a standardized path coefficient of β = 0.567 (SE = 0.102, z = 5.52, *p* < 0.001), suggesting that higher knowledge is associated with stronger personal intention to use or consume *Spirulina*. The direct effect of knowledge on social motivation/influence (F3) was positive but not statistically significant (β = 0.133, SE = 0.083, z = 1.78, *p* = 0.075), indicating that knowledge alone has a limited direct impact on social motivation. Personal intention/behaviour, however, strongly predicts social motivation (F3; β = 0.821, SE = 0.107, z = 8.59, *p* < 0.001), demonstrating that individual intentions largely drive social and motivational outcomes.

Model fit indices indicated an acceptable but not ideal fit. The chi-square test was significant (χ^2^ = 389.86, *df* = 132, *p* < 0.001), as often occurs with moderate sample sizes. Additional fit indices showed a Comparative Fit Index (CFI) of 0.887, Tucker–Lewis Index (TLI) of 0.868, Goodness-of-Fit Index (GFI) of 0.911, Adjusted GFI of 0.873, Root Mean Square Error of Approximation (RMSEA) of 0.120, and Standardized Root Mean Square Residual (SRMR) of 0.053. The SRMR indicates an acceptable fit, whereas the elevated RMSEA suggests some model misspecification, potentially due to residual correlations or omitted paths.

Multi-group SEM analyses indicated no significant moderating effects of gender or age on the structural relationships. The scaled chi-squared difference tests showed invariance across groups for gender (Δχ^2^ = 1.28, Δ*df* = 3, *p* = 0.734) and age (Δχ^2^ = 1.65, Δ*df* = 3, *p* = 0.649), suggesting that the model is stable across both demographic factors.

Although some descriptive differences in path coefficients were observed, for example, younger respondents and females showed slightly stronger direct effects of F1 on F3, while older respondents and males relied more on the mediated path through F2, these differences were not statistically significant. The structural relationships are consistent across age and gender, indicating that demographic factors do not meaningfully alter the proposed model.

The SEM results support the hypothesized directional relationships among knowledge, intention, and social motivation, with knowledge significantly shaping behaviour, which in turn strongly influences social motivation.

### 3.13. Mediator Effect

The estimated indirect effect (F1 → F2 → F3) was significant (β = 0.465, SE = 0.112, z = 4.62, *p* < 0.001), and the total effect of knowledge on social motivation, including both direct and indirect paths, was also significant (β = 0.598, SE = 0.118, z = 5.65, *p* < 0.001). These results indicate a full mediation, whereby knowledge influences social motivation primarily through its effect on personal intention, highlighting the pivotal role of individual intention as a mediator between knowledge and socially oriented motivational outcomes.

### 3.14. Weighted Least Squares Mean-Adjusted Estimator in SEM

The measurement model demonstrated good internal consistency and convergent validity. Composite reliability (CR) values were 0.789 for F1, 0.951 for F2, and 0.921 for F3, exceeding the recommended threshold of 0.70. Standardized factor loadings were generally strong, ranging from 0.421 (Q6, F1) to 0.969 (Q12, F2), with most items above 0.70, confirming that the indicators reliably measure their respective latent constructs. Average Variance Extracted (AVE) values were similarly satisfactory, supporting convergent validity.

Discriminant validity assessed via the Heterotrait–Monotrait ratio (HTMT) [48,49] indicated values of 1.00 for all construct pairs (F1–F2, F1–F3, F2–F3), exceeding the recommended threshold of 0.85, suggesting substantial overlap between constructs. This may reflect conceptual proximity among the latent variables, causing a potential reduced discriminant validity and blurred the distinction between these constructs. It is consistent with the conceptual proximity of knowledge, personal intention, and social motivation in the KAP framework, and should be considered when interpreting these findings. In this context, the observed HTMT values were not interpreted as evidence of model inadequacy, but rather as an expected outcome of theoretically overlapping constructs within a unified behavioural continuum.

Comparing estimation methods, the Weighted Least Squares Mean- and Variance-adjusted estimator (WLSMV), appropriate for ordinal Likert-scale indicators, demonstrated superior model fit and was therefore retained as the final structural equation model for interpretation. The WLSMV model achieved excellent fit indices (CFI = 0.999, TLI = 0.998, RMSEA = 0.059, SRMR = 0.056), whereas the MLR model showed only moderate fit (CFI = 0.887, TLI = 0.868, RMSEA = 0.120, SRMR = 0.053). Accordingly, all structural interpretations, including direct and indirect effects, were based exclusively on the WLSMV solution, while the MLR results are reported solely for comparative and robustness purposes.

The elevated RMSEA observed in the MLR model likely reflects sensitivity to distributional assumptions and model complexity when applied to ordinal data. This discrepancy further supports the appropriateness of the WLSMV estimator for the present dataset. Given the ordinal nature of the data, the WLSMV estimator was considered the primary analytical solution, while MLR was retained only for comparative robustness assessment and was not used for inferential interpretation. The improved fit indices obtained using the WLSMV estimator, which is more robust to non-normality and suitable for ordinal data, further indicate that estimation method plays a role in the observed discrepancies. Therefore, while the model captures the main structural relationships, these results should be interpreted with consideration of possible omitted effects and estimation limitations.

Structural paths in the WLSMV model indicated that F1 strongly predicted F2 (β = 0.595, *p* < 0.001), and F2 strongly predicted F3 (β = 0.877, *p* < 0.001). The direct effect of F1 on F3 was small and non-significant (β = 0.074, *p* = 0.214), suggesting a mediated relationship through F2.

Explained variance was substantial, with R^2^ = 0.354 for F2 and R^2^ = 0.851 for F3, indicating that the model accounted for a large proportion of variance in the endogenous constructs. Individual items were also well-explained, with R^2^ values up to 0.969 for Q12 (F2) and 0.929 for Q18 (F3).

The WLSMV SEM provides a robust representation of the latent structure, demonstrating strong reliability, convergent validity, and excellent model fit, while highlighting the mediating role of F2 in the relationship between F1 and F3.

These results all point to the same thing: intention is a key link between knowledge and behaviour. This shows that, in Serbia, just increasing consumer knowledge is not enough to get people to eat algae-based foods and motivational and behavioural factors must also be addressed, contrary to the results of the study conducted in Spain [14]. Previous study conducted in Norway showed that environmental concern has significant positive effect on consumers’ attitude and purchase intentions towards microalgae-based food [24]. This conclusion is different than conclusion presented in this study, where health concerns (F2) represent the major driving factor for consumers’ willingness to drink juice enriched with *Spirulina*. Regarding beverages, a previous study by Rombach and Dean [15] showed that emphasising both sustainability and nutritional advantages of microalgae can increase appeal of this type of products.

### 3.15. Consumers’ Survey on Acceptability of Juices Enriched with Spirulina

A total of 110 consumers participated in a preference test that included a hedonic assessment. In this test, consumers rated the acceptability of six juice samples based on appearance, odour, texture, taste, and overall acceptability, using a seven-point hedonic scale. Additionally, consumers ranked the products from 1 (most preferred) to 6 (least preferred).

The scores for the acceptability ratings and rankings of the samples are presented in Table 4. Sample SC2 received the highest ranking, with a score of 2.57 ± 1.32, while its overall acceptability was 5.93 ± 1.03. Notably, the overall acceptability of SC2 did not differ significantly (*p* ≤ 0.05) from that of SC3. Interestingly, both sour cherry juice samples with *Spirulina* added received better rankings and overall acceptability scores compared to plain sour cherry juice. This contrasts with the findings for tomato juice, where the addition of *Spirulina* resulted in lower rankings and overall acceptability scores, which decreased with the increasing amount of *Spirulina* added. Specifically, sample T3 received lower scores for nearly all assessed parameters compared to T2, except for appearance and odour.

The appeal of the enriched sour cherry juice’s appearance to consumers can be related to the natural colour of the juice, which is red-purple due to the presence of anthocyanins, making it more suitable for the addition of blue *Spirulina* powder than tomato juice colour (naturally red–orange). Better consumers’ reaction to sour cherry juice in comparison to tomato juice as a matrix for *Spirulina* addition may be due to its higher acidity and stronger flavour. Although tomato juice is traditionally used for sauces for fish, sour cherry juice was shown to be more potent for fishy odour and taste masking. Namely, the addition of citrus juice and other products that contain organic acids, as well as strong flavours, are well-known techniques for masking the fishy flavour in aquatic products in daily cooking and ready-to-eat food [11,50].

Sample SC1 was noted for having the highest acceptability ratings in terms of appearance (6.13 ± 1.12), odour (5.97 ± 1.25), and texture (5.76 ± 1.14). However, SC2 and SC3 were found to be superior in taste, likely influencing a more favourable overall impression among consumers. These differences in acceptability can be attributed to variations in sensory properties, determined both instrumentally and by the panel of trained assessors, as outlined in a previous publication by Cvetković et al. [18]. The success of SC2 and SC3 appears partly driven by strong visual appeal, suggesting that consumers may tolerate minor taste limitations in favour of appearance, as reflected by their high overall acceptability ratings despite moderate taste scores. Additionally, this is a very important finding, as taste has been shown in previous studies to be a crucial factor in purchase decisions for *Spirulina*-containing products [12].

### 3.16. Penalty Analysis on Acceptability of Juices Enriched with Spirulina

The results of the JAR-based penalty analysis are presented for colour, odour, texture, taste, and overall acceptability (Table 5). No statistically significant penalties (*p* > 0.05) were observed for any of the evaluated sensory attributes, indicating that deviations from the just-about-right level did not significantly affect consumer hedonic perception within the studied sample.

Although some numerical trends in mean drop values were observed, particularly for the “too much” category in taste (mean drop = 0.410) and texture (mean drop = 0.276), these differences were not statistically significant. Similarly, colour and odour exhibited relatively small mean deviations across JAR categories, suggesting a generally stable sensory acceptance profile.

The absence of significant penalties indicates that consumers demonstrated a relatively broad acceptance range for the evaluated product, without strong sensitivity to minor deviations in attribute intensity. This may suggest that the incorporation of *Spirulina* did not introduce critical sensory limitations affecting product acceptability. From a product development perspective, these findings imply that formulation adjustments within the tested range are unlikely to significantly alter overall consumer liking, which is advantageous for further optimization and potential market introduction.

The results indicate that although a considerable proportion of consumers perceived certain sensory attributes, particularly taste, texture, and odour, as too intense, the magnitude of penalty effects remained relatively low. This suggests a broad acceptance window for the *Spirulina*-enriched product, with only minor reductions in hedonic liking associated with deviations from the JAR level.

### 3.17. Strengths and Limitations of the Study

The present study employed a convenience sampling approach based on voluntary participation, which may introduce selection bias. As a result, the sample was not fully representative of the general population, as evidenced by the predominance of female respondents and individuals with higher educational attainment. The principal limitation of the present study is the relatively small sample size (*n* = 136), which may constrain the statistical power of the analyses and limit the generalizability of the findings. A larger sample would enable more precise parameter estimation, increase the robustness of inferential statistics, and allow for more detailed stratified analyses across relevant sociodemographic variables. Consequently, the external validity of the results should be interpreted with caution.

In addition, although the sample size (*n* = 136) may be considered modest for Structural Equation Modelling (SEM), it falls within the commonly recommended range for models of moderate complexity. Methodological guidelines suggest minimum sample sizes of 100–200 observations or a ratio of 5–10 cases per estimated parameter, which was satisfied in this study. The obtained model fit indices and stability of parameter estimates further indicate that the sample size was sufficient to detect the principal relationships of interest. Nevertheless, it should be acknowledged that the statistical power remains limited for detecting smaller effect sizes and for conducting more complex analyses, such as multi-group comparisons.

The methodological strength of the study is reflected in the consistency and reliability of the applied statistical procedures. The obtained results demonstrated satisfactory model performance and internal coherence, indicating that, despite the moderate sample size, the main relationships among the investigated variables were sufficiently stable to support the principal conclusions. The direction and magnitude of the identified associations were theoretically plausible and statistically substantiated within the examined population.

Future investigations should therefore be designed with larger and more heterogeneous samples, ideally incorporating respondents from multiple countries and diverse cultural, socioeconomic, and dietary backgrounds. The inclusion of cross-national samples would enable more robust comparative analyses and improve the external validity of the findings. In particular, such an approach would allow for the examination of potential cultural moderators influencing the relationships between consumers’ general knowledge of algae, their specific awareness of *Spirulina*, their attitudes and intentions toward algae-based products, and their actual consumption behaviour. Cross-cultural data would also facilitate the assessment of structural differences in consumer perception models and the stability of predictive relationships across distinct market environments.

Further investigations should also focus on consumer preferences for specific product categories, such as juices enriched with *Spirulina*, by evaluating sensory expectations, perceived health benefits, price sensitivity, and trust in novel food ingredients. The application of advanced multivariate techniques (such as structural equation modelling or multi-group analysis) would enable a more detailed identification of determinants of purchase intention and actual buying behaviour. Such research would contribute to a more comprehensive understanding of acceptance drivers, potential barriers, and market segmentation strategies across different populations and geographic regions.

## 4. Conclusions

In this study, the relationship between knowledge, attitudes, and practices regarding the consumption of *Spirulina*-enriched fruit and vegetable juices was analysed using a two-sided approach: structural equation modelling and consumer preference evaluation. The answers in the survey showed moderate to high correlations, indicating that exploratory factor analysis (EFA) should be performed. The EFA results suggest a three-dimensional structure of the questionnaire, encompassing knowledge, personal intention/behaviour, and social motivation, and they were further used for structural equation modelling (SEM). The SEM results pointed out that, in this study conducted in Serbia, the direct effect of knowledge on social motivation was positive but not statistically significant, indicating the limited direct impact of knowledge on social motivation. However, personal behaviour strongly predicts social motivation, stressing the importance of individual intentions. Namely, perceived nutritional value and health benefits were shown to be the main factors for consumers’ willingness to drink *Spirulina*-enriched juice.

In general, the result of the consumer preference analysis showed that the juices containing sour cherry and *Spirulina* achieved better sensory acceptance and ranking than those containing tomato. The sour cherry matrix masked the characteristic taste and odour of *Spirulina* more effectively than tomato. This is likely due to the higher acidity and pronounced fruity flavour of sour cherries, which balance *Spirulina*’s umami and earthy notes, whereas tomatoes have a milder flavour and are less effective at concealing these sensory characteristics. Taste was identified as the main factor influencing overall acceptability for all juices, followed by texture and odour as secondary factors, and finally colour as a minor factor. These findings indicate that the successful integration of *Spirulina* into fruit and vegetable juices is related to the optimisation of the product’s taste and the importance of the product matrix for achieving consumer acceptance.

## Figures and Tables

**Table 1:** Questions in survey analysis.

**Sociodemographic Variables (A1–A5):**
A1: Gender
A2: Age
A3: Highest level of completed education
A4: Current status (e.g., student, employed, unemployed)
A5: Monthly income (national average in August 2023: 725 EUR)
**Knowledge Items (Q1–Q5):**
Q1: I am aware that algae are used in everyday human nutrition.
Q2: I am aware that consuming algae is beneficial for maintaining good health.
Q3: I know that algae use solar energy to accumulate nutrients.
Q4: I have heard that *Spirulina* algae are known as “superfood.”
Q5: I would know how to prepare a meal or drink with algae.
**Intention Items (Q6–Q12):**
Q6: I have tried algae in food or drink.
Q7: I consider consuming *Spirulina* algae to be beneficial, but I would not use it personally.
Q8: I consider consuming *Spirulina* algae beneficial, but I would not consume it in food or drink.
Q9: I consider consuming *Spirulina* algae beneficial and would like to include it in my daily diet for health reasons.
Q10: I would use *Spirulina* algae because it contains high protein content and can partially replace meat.
Q11: I would use *Spirulina* algae because it contains high protein and all essential amino acids.
Q12: I would use *Spirulina* algae because it contains polyunsaturated fatty acids beneficial for maintaining good health.
**Behavioural/Belief Items (Q13–Q22):**
Q13: I would recommend *Spirulina* algae in the diet because it is beneficial for individuals with malnutrition.
Q14: I would consume *Spirulina* algae because it reduces disease risk and positively affects health.
Q15: I am attracted to using algae because they are self-sustainable.
Q16: I am attracted to using algae because they have high nutritional value.
Q17: I am attracted to using algae because it is trendy to consume them.
Q18: I am attracted to using algae because of health benefits.
Q19: An affordable price would motivate me to consume food or drinks in a catering setting containing added *Spirulina* algae.
Q20: Positive opinions from others about using *Spirulina* algae would motivate me to consume food or drinks containing it in a catering setting.
Q21: Positive health effects would motivate me to consume food or drinks with added *Spirulina* algae in a catering setting.
Q22: Consuming *Spirulina* algae in food or drink is beneficial and may be enjoyable, as it changes the colour of the food or drink to blue or green due to the algae pigment.

**Table 2:** Descriptive statistics for Q1–Q22.

Variable	Mean	S.D.	Median	Trimmed	Min	Max	Skew	Kurtosis	S.E.
Q1	3.68	1.42	4	3.85	1	5	−0.67	−0.94	0.12
Q2	3.89	1.31	4	4.08	1	5	−0.92	−0.41	0.11
Q3	4.07	1.17	5	4.26	1	5	−1.03	0.08	0.10
Q4	3.49	1.42	4	3.61	1	5	−0.47	−1.05	0.12
Q5	2.30	1.28	2	2.15	1	5	0.60	−0.73	0.11
Q6	2.71	1.69	2	2.65	1	5	0.29	−1.63	0.14
Q7	2.58	1.27	3	2.48	1	5	0.34	−0.82	0.11
Q8	2.59	1.29	3	2.49	1	5	0.36	−0.88	0.11
Q9	3.35	1.26	4	3.44	1	5	−0.53	−0.72	0.11
Q10	3.16	1.33	3	3.20	1	5	−0.26	−1.03	0.11
Q11	3.51	1.29	4	3.64	1	5	−0.56	−0.69	0.11
Q12	3.51	1.29	4	3.64	1	5	−0.58	−0.66	0.11
Q13	3.44	1.22	3	3.55	1	5	−0.44	−0.54	0.10
Q14	3.66	1.21	4	3.81	1	5	−0.69	−0.26	0.10
Q15	3.20	1.33	3	3.25	1	5	−0.31	−1.01	0.11
Q16	3.31	1.31	3	3.38	1	5	−0.42	−0.88	0.11
Q17	2.33	1.32	2	2.17	1	5	0.58	−0.79	0.11
Q18	3.57	1.33	4	3.70	1	5	−0.69	−0.62	0.11
Q19	3.46	1.27	4	3.56	1	5	−0.46	−0.80	0.11
Q20	3.35	1.36	4	3.43	1	5	−0.46	−1.00	0.12
Q21	3.67	1.29	4	3.83	1	5	−0.80	−0.43	0.11
Q22	3.28	1.39	3	3.35	1	5	−0.27	−1.15	0.12

**Table 3:** Exploratory Factor Analysis.

Variable	ML1	ML3	ML2
Q1			0.778
Q2			0.820
Q3			0.607
Q4			0.638
Q6			0.464
Q9	0.538		
Q10	0.751		
Q11	0.987		
Q12	0.967		
Q13	0.540		
Q14	0.494		
Q15	0.627		
Q16	0.612		
Q18	0.475		
Q19		0.859	
Q20		0.675	
Q21		0.773	
Q22		0.699	

**Table 4:** Liking ratings for individual sensory characteristics.

Sample	Acceptability Ratings					Ranking of the Samples
	Appearance	Odour	Texture	Taste	Overall	
SC1	6.13 ± 1.12 ^a^	5.97 ± 1.25 ^a^	5.76 ± 1.14 ^a^	5.58 ± 1.50 ^bc^	5.80 ± 1.12 ^ab^	2.97 ± 1.67 ^a^
SC2	5.68 ± 1.45 ^b^	5.55 ± 1.42 ^b^	5.57 ± 1.29 ^a^	5.95 ± 1.38 ^ab^	5.93 ± 1.03 ^a^	2.57 ± 1.32 ^a^
SC3	5.58 ± 1.48 ^b^	5.36 ± 1.53 ^b^	5.62 ± 1.33 ^a^	5.99 ± 1.19 ^a^	5.93 ± 1.11 ^a^	2.80 ± 1.75 ^a^
T1	5.61 ± 1.40 ^b^	5.27 ± 1.60 ^b^	5.52 ± 1.41 ^a^	5.57 ± 1.57 ^bc^	5.52 ± 1.45 ^b^	3.41 ± 1.52 ^b^
T2	3.55 ± 1.75 ^c^	4.55 ± 1.59 ^c^	5.11 ± 1.44 ^b^	5.27 ± 1.58 ^c^	5.00 ± 1.33 ^c^	4.15 ± 1.43 ^c^
T3	3.73 ± 1.81 ^c^	4.68 ± 1.50 ^c^	5.00 ± 1.48 ^b^	5.21 ± 1.65 ^c^	4.93 ± 1.45 ^c^	4.30 ± 1.67 ^c^

Data are expressed as means (*n* = 110) ± standard deviations. Lowercase letters indicate statistically significant difference at *p* ≤ 0.05.

**Table 5:** Penalty analysis of sensory attributes and their impact on hedonic acceptability of *Spirulina*-enriched product.

Var.	Level	Freq.	Sum	Mean Drops	Stand. Diff.	*p*-Value	Penalties	Stand. Diff.	*p*-Value
Colour	Too little	56	247	−0.054	−0.132	0.990			
	JAR	14	61				0.065	0.135	0.8930
	Too much	40	165	0.232	0.608	0.816			
Odour	Too little	21	93	0.054					
	JAR	29	130				0.248	0.678	0.4992
	Too much	60	250	0.316	0.917	0.361			
Texture	Too little	18	80	0.056					
	JAR	16	72				0.234	0.511	0.6103
	Too much	76	321	0.276	0.642	0.522			
Taste	Too little	17	80	−0.122					
	JAR	12	55				0.318	0.614	0.5405
	Too much	81	338	0.410	0.852	0.396			
Overall acceptability	Too little	21	97	−0.169					
	JAR	20	89				0.1833	0.438	0.6623
	Too much	69	287	0.291	0.7720	0.4418			

## Data Availability

The original contributions presented in the study are included in the article/Supplementary Materials, further inquiries can be directed to the corresponding author.

## Supplementary Materials

The following supporting information can be downloaded at: https://www.mdpi.com/article/10.3390/nu18081309/s1, File S1: Survey questions.docx; File S2: Survey data.csv; File S3: Consumers test data.csv; File S4: Not normal transformation.txt; File S5: SEM model.txt.
