# Supplementary material for: Relationship Between Knowledge, Attitudes, and Practices for the Consumption of Spirulina-Enriched Fruit and Vegetable Juices: Structural Equation Modelling and Consumers’ Preference Evaluation Approach
Source: Nutrients. 2026 Apr 21;18(8):1309. doi: 10.3390/nu18081309 (PMC13119012; doi:10.3390/nu18081309)
Supplement: Supplementary file 1 [file nutrients-18-01309-s001.zip › File S1 - Survey questions.pdf]

Questionnaire on Knowledge, Attitudes, and Behavioral Intentions Regarding Spirulina Consumption

Section A. Sociodemographic Characteristics

A1. Gender

A2. Age

A3. Highest level of education attained

A4. Current employment status

A5. Monthly income

(Note: The national average monthly income in Serbia in August 2023 was 85,066 RSD.)

Section K. Knowledge About Algae

Q1. I am aware that algae are used in everyday human nutrition.

Q2. I am aware that the consumption of algae is beneficial for maintaining good health.

Q3. I know that algae use solar energy to accumulate nutrients.

Q4. I have heard that the alga Spirulina is also referred to as a "superfood."

Q5. I would know how to prepare a meal or beverage containing algae.

Section I. Personal Experience and Intentions

Q6. I have tried algae in food or beverages.

Q7. I believe that consuming Spirulina is beneficial; however, I personally would not use it.

Q8. I believe that consuming Spirulina is beneficial; however, I would not consume it in food or beverages.

Q9. I believe that consuming Spirulina is beneficial and I would like to include it in my daily diet for health reasons.

Q10. I would use Spirulina because it contains a high level of protein and can serve as a partial substitute for meat.

Q11. I would use Spirulina because it contains a high level of protein and provides all essential amino acids.

Q12. I would use Spirulina because it contains polyunsaturated fatty acids that are beneficial for maintaining good health.

Section B. Beliefs and Behavioral Motivation

Q13. I would recommend Spirulina in the diet because it may be beneficial for individuals suffering from malnutrition.

Q14. I would consume Spirulina because it may reduce the risk of disease and contribute positively to health.

Q15. I am attracted to the use of algae because they are environmentally sustainable.

Q16. I am attracted to the use of algae because of their high nutritional value.

Q17. I am attracted to the use of algae because their consumption is currently considered fashionable or trendy.

Q18. I am attracted to the use of algae because of their potential health benefits.

Q19. An affordable price would motivate me to consume food and beverages in food service establishments that contain added Spirulina.

Q20. Positive opinions of others regarding the use of Spirulina would motivate me to consume food and beverages in food service establishments containing added algae.

Q21. Positive health effects would motivate me to consume food and beverages with added Spirulina in food service establishments.

Q22. Consuming Spirulina in food or beverages may be beneficial and enjoyable, as foods or drinks can change color to blue or green due to the pigments present in the algae.

Response format:

All statements (Q1–Q22) were evaluated using a five-point Likert scale, ranging from 1 – strongly disagree to 5 – strongly agree.
